# Effect of Extracellular Vesicles Derived From Tumor Cells on Immune Evasion

**DOI:** 10.1002/advs.202417357

**Published:** 2025-02-03

**Authors:** Xuanfan Liu, Kenneth K.W. To, Qinsong Zeng, Liwu Fu

**Affiliations:** ^1^ State Key Laboratory of Oncology in South China Guangdong Provincial Clinical Research Center for Cancer Collaborative Innovation Center for Cancer Medicine Guangdong Esophageal Cancer Institute Sun Yat‐sen University Cancer Center Guangzhou 510060 P. R. China; ^2^ Department of Urology The First Affiliated Hospital of Sun Yat‐sen University Guangzhou 510080 P. R. China; ^3^ School of Pharmacy The Chinese University of Hong Kong Hong Kong 999077 P. R. China; ^4^ Guangxi Hospital Division of The First Affiliated Hospital Sun Yat‐sen University Nanning 530025 P. R. China

**Keywords:** extracellular vesicles, immune evasion, immunotherapy, targeted therapies, tumor microenvironment

## Abstract

The crosstalk between immunity and cancer in the regulation of tumor growth is considered a hallmark of cancer. Antitumor immunity refers to the innate and adaptive immune responses that regulate cancer development and proliferation. Tumor immune evasion represents a major hindrance to effective anticancer treatment. Extracellular vesicles (EVs) are nano‐sized and lipid‐bilayer‐enclosed particles that are secreted to the extracellular space by all cell types. They are critically involved in numerous biological functions including intercellular communication. Tumor‐derived extracellular vesicles (TEVs) can transport a variety of cargo to modulate immune cells in the tumor microenvironment (TME). This review provides the latest update about how tumor cells evade immune surveillance by exploiting TEVs. First, the biogenesis of EVs and the cargo‐sorting machinery are discussed. Second, how tumor cells modulate immune cell differentiation, activation, and function via TEVs to evade immune surveillance is illustrated. Last but not least, the novel antitumor strategies that can reverse immune escape are summarized.

## Graphical abstract

Immunity and cancer interplay are central to tumor progression. This review explores how tumor cells exploit extracellular vesicles (EVs) to orchestrate immune evasion. Tumor‐derived EVs (TEVs) transport immunomodulatory cargo to shape immune differentiation, activation, and function. EV biogenesis, sorting mechanisms, immune regulation, and promising antitumor strategies for reversing immune escape are presented.

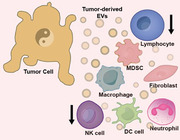

## 1 Introduction

Extracellular vesicles (EVs) are nano‐sized and lipid‐bilayer‐enclosed particles that are released by all cell types.^[^ ^1^ ^]^ Initially, upon their discovery, EVs were considered as cellular waste vesicles or particles generated by cellular damage.^[^ ^2^, ^3^ ^]^ With accumulating research in EV biology, EVs are now known to be secreted by living cells and they are critically involved in numerous biological functions including cell‐to‐cell communication.^[^ ^4^ ^]^ The cargo of EV consists of proteins, mRNAs, miRNAs, lipids, metabolites, and other biologically active molecules. These components are transferred via the EVs and interact with neighboring cells to alter their biological properties.^[^ ^4^, ^5^ ^]^ Notably, cancer cells have been recognized as cells that can educate surrounding cells, alter their function, and finally remold TME. Obviously, TEVs are good tools for cancer cells to complete these processes.

Over the past decades, TEVs have been reported to mediate intercellular crosstalk within the TME, leading to tumor invasion, growth, metastasis, angiogenesis, immune escape, and drug resistance.^[^ ^6^, ^7^, ^8^ ^]^ Importantly, numerous studies have demonstrated that TEVs can facilitate remote communication via blood circulation, underscoring their significant role in remodeling the systemic immune environment by tumors.^[^ ^9^ ^]^ Moreover, compelling evidence indicates that TEVs can inhibit anti‐tumor responses by targeting specific receptors or ligands expressed on immune cells.^[^ ^10^ ^]^ It has been shown that TEVs can deliver numerous immunosuppressive molecules, such as PD‐L1, Fas‐L, and TGF‐β, to directly target natural killer (NK) cells and effector CD8+ T cells, thereby suppressing antitumor immunity.^[^ ^11^ ^]^ In addition, TEVs have also been reported to regulate tumor immune evasion, growth, neovascularization, invasion, and metastasis within the TME by shuttling between donor cells and targeted cells.^[^ ^12^, ^13^, ^14^, ^15^, ^16^ ^]^ In recent years, growing number of studies have revealed that the immunosuppressive effect induced by TEVs can be mediated through a variety of tumor infiltrating immune cells, such as NK cells, tumor associated macrophages (TAMs), dendritic cells (DCs), myeloid‐derived suppressor cells (MDSCs), αβ T cells, γδ T cells, and neutrophils.^[^ ^17^, ^18^, ^19^ ^]^  As immunotherapy has emerged as a cutting‐edge treatment for eliminating malignant tumor cells, elucidating the effects and mechanisms of TEVs on immune evasion can help researchers understand the underlying reason for drug resistance, and aid in the development of more effective anti‐tumor strategies.

Therefore, in this review, we collated evidence demonstrating that TEVs alter tumor immunity by modulating the antitumor function of immune cells.  Additionally, recent advances in EV‐based anti‐tumor immunotherapy strategies are discussed.

## 2 Overview of EVs

### 2.1 Classification of EVs

EVs can be divided into three main subtypes, exosomes, microvesicles (MVs), and apoptotic bodies, according to the mechanisms of biogenesis and secretion, size, cargo content, and their biological functions.^[^ ^1^ ^]^  The International Society for Extracellular Vesicles (ISEV) has recently updated its guideline called “Minimal Information for Studies of Extracellular Vesicles 2023 (MISEV 2023)” to precisely define the organellar origin of the various EV subtypes.^[^ ^1^ ^]^

Exosomes, MVs, and apoptotic bodies can be differentiated by size.  Exosomes are the smallest EVs with a size range of 30–150 nm. MVs are considered small/large EVs, that are 100–1000 nm in diameter.  Apoptotic bodies are larger EVs with a size range of 1000–5000 nm.  More recently, a few newer EV subtypes, including exophers, large oncosomes, migrasomes, and some membrane‐less, non‐vesicular extracellular particles (exomeres and supermeres), have been introduced.^[^ ^20^, ^21^, ^22^, ^23^, ^24^ ^]^  However, the specific physiological roles of these new EV subtypes and their precise biogenesis mechanisms have not been clearly elucidated.  It is noteworthy that most current studies on TEVs focus on small EVs (sEVs, including small ectosomes and exosomes) with diameters less than 200 nm.  Therefore, elucidating the biogenesis and the cargo‐sorting mechanisms of sEVs, especially exosomes, could provide a foundation for investigating the effects of TEVs in tumor immunomodulation and possibly designing novel and effective therapeutic strategies.

### 2.2 Biogenesis of EVs

**Figure 1:**
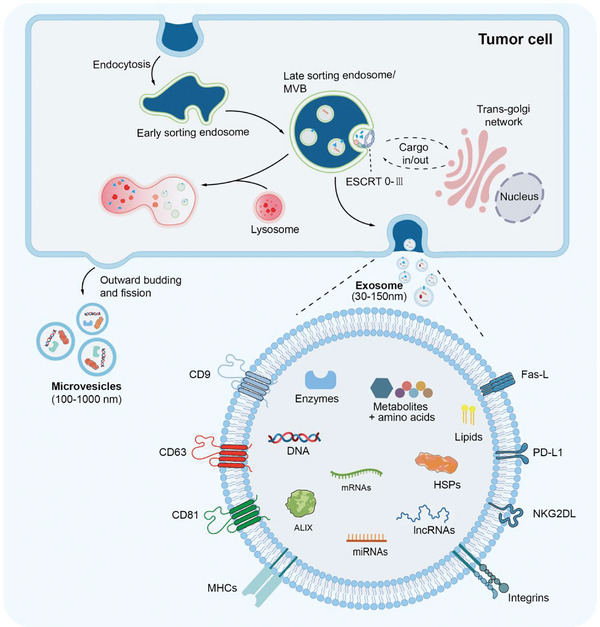
Biogenesis and cargo of EVs. EVs (exosomes being the most extensively characterized one) are secreted into the extracellular space when multivesicular bodies (MVBs) fuse with the plasma membrane. The process starts with endocytosis. Multiple mechanisms drive the inward budding of the plasma membrane to form the early endosomes. Various cargoes are incorporated into the early endosomes to generate MVBs. Finally, the mature MVBs fuse either with the lysosomes to be degraded or with the plasma membrane to release the exosomes. Exosomes are known to contain a range of different cargoes, including nucleic acids, proteins, lipids, and metabolites. Different receptors such as Fas‐L, PD‐L1, CD63, and NKG2DL are expressed on the exosome surface to regulate immune cells in the TME.

While EVs are released by all cell types, the biogenesis of the three major subtypes is different.  Exosomes are secreted to the extracellular space via the endosomal pathway.  On the other hand, MVs originate directly from the plasma membrane.  Apoptotic bodies are secreted from the membrane blebs of cells undergoing apoptosis.  The sequential steps involved in the biogenesis of exosomes have been clearly elucidated (**Figure** **1**).^[^ ^5^ ^]^  First, the cytoplasmic membrane undergoes the first invagination to form early sorting endosomes (ESEs), which contain soluble factors and membrane proteins related to the extracellular environment.  Under certain circumstances, these newly formed ESEs may merged directly with pre‐existing ESEs.  Second, ESEs will further mature into late‐sorting endosomes (LSEs) and ultimately give rise to intracellular multivesicular vesicles (MVBs), also known as multivesicular endosomes.^[^ ^25^, ^26^ ^]^  Afterward, the formation of intraluminal vesicles (ILVs) is induced via the inward budding of the endosomal membrane. Therefore, the production of ILVs is constituted by a double invagination of the plasma membrane.  Then, MVBs will fuse with the plasma membrane and ILVs will be secreted into extracellular space as exosomes.^[^ ^5^ ^]^  Meanwhile, MVBs can also bind to the lysosome and the content will be degraded by lysosomal enzymes.

Mechanistically, the production of exosomes involves a complex molecular regulatory network, where a variety of molecules can synergistically or independently regulate the invagination and cleavage of the plasma membrane, and also the cargo sorting process (Figure 1).^[^ ^5^ ^]^  Numerous studies have shown that multiple molecular mechanisms contribute to the biogenesis of exosomes. The ESCRT (endosomal sorting complexes required for transport) refers to the classic pathway in exosome biogenesis, which works gradually and involves multiple ESCRT components (ESCRT 0‐III).^[^ ^27^ ^]^  Through the combined action of these components, ubiquitinated cargo can be captured.^[^ ^28^, ^29^ ^]^  Interestingly, it has been shown that ceramides and transmembrane proteins can independently regulate the biogenesis of exosomes via different pathways.^[^ ^30^, ^31^, ^32^ ^]^  After the formation of MVBs, they may communicate with other organelles, such as the endoplasmic reticulum, trans‐Golgi network, and mitochondria, to promote the maturation of MVBs.^[^ ^33^, ^34^, ^35^, ^36^, ^37^ ^]^  Studies have shown that Rabs, a family of GTPases, regulate the biogenesis, fate, and transportation of MVBs.^[^ ^38^, ^39^, ^40^, ^41^ ^]^  When mature MVBs are transported near the plasma membrane, they will fuse with membrane via a sensitive factor attachment protein receptor (SNARE) complex, subsequently releasing exosomes.^[^ ^42^, ^43^ ^]^

In contrast, MVs (another EV subtype) are generated by the outward budding and fission of cell membrane (Figure 1).^[^ ^5^, ^44^ ^]^ MVs have also been reported to play an important role in the regulation of tumor immunity.^[^ ^45^, ^46^, ^47^ ^]^ On the other hand, apoptotic bodies are produced specifically during cell apoptosis.^[^ ^48^ ^]^  When cells undergo apoptosis, the cellular materials, including DNA fragments, cytosolic fractions, and degraded proteins, are encapsulated into membrane‐bound vesicles, subsequently generating apoptotic bodies.^[^ ^49^ ^]^

### 2.3 The Cargo Delivered in EVs

The communication between different cells, tissues, and organs plays critical roles both in the maintenance of body homeostasis and in disease development.  EVs are well‐established mediators of intercellular cross‐talk by transferring mRNAs, microRNAs, proteins, and metabolites from donor to recipient cells.^[^ ^50^, ^51^ ^]^ It is noteworthy that the contents of EVs exhibit cellular diversity and generally mirror the physiological or pathological condition of the parent cells.^[^ ^5^, ^26^ ^]^  However, it has also been suggested that the content of EVs is not simply a reflection of the cell content of origin, but that specific cargos are selectively packaged or sorted into EVs.^[^ ^52^ ^]^  Some biomarker molecules such as CD9, CD63, CD81, TSG101 (tumor susceptibility gene 101), ALIX (apoptosis‐linked gene 2‐interacting protein X ), and HSP70 (heat shock protein 70), are constitutively expressed on EVs (Figure 1).^[^ ^53^ ^]^  Consistently, TEVs can carry a variety of specific immunomodulatory proteins, such as tumor antigens  PD‐L1, Fas‐L, heat shock proteins, galectin‐9, and TGF‐β.  TEVs also contain functional nucleic acids, such as microRNAs (miRNAs), mRNA, circular RNAs (circRNAs), long noncoding RNAs (lncRNAs), and DNA.  Notably, EVs exhibit significant heterogeneity, with EVs derived from different tumor types highly expressing molecular markers that reflect the characteristics of their parent cells.^[^ ^54^ ^]^ For instance, EVs originating from breast cancer demonstrate elevated levels of HER2,^[^ ^55^ ^]^ those from pancreatic cancer show increased expression of EGFR and CA19‐9,^[^ ^56^ ^]^ and prostate cancer‐derived EVs are enriched with the mRNAs of PCA3, ERG, and SPDEF.^[^ ^57^ ^]^ Furthermore, additional studies have undertaken the characterization of tumor‐derived EVs (TEVs) to identify biological targets indicative of their distinct features. For example, Ko et al. have demonstrated that CD147 serves as a characteristic molecule of TEVs enriched with miRNA.^[^ ^58^ ^]^

### 2.4 The Routes of EVs Interacting with Target Cells

EVs derived from different cell types can interact with the target cells in multiple ways. Once EVs are secreted into extracellular environment, they can induce intercellular signaling via ligand receptor binding, endocytosis, or membrane fusion.^[^ ^59^, ^60^ ^]^ Ligand‐receptor binding is based on the high affinity between them. Interestingly, compared to soluble ligands, researchers have found that EVs can trigger the localized aggregation of membrane receptors at contact sites during their interaction with membrane receptors. This aggregation facilitates protein–protein interactions, elucidating the unique structural advantages of EV‐mediated signal transduction.^[^ ^61^ ^]^ However, unlike the principle of affinity, the mechanisms by which target cells recognize and uptake EVs via endocytosis or membrane fusion still require further investigation. The internalization of EVs by target cells can be mediated by various molecules, including tetraspanins, caveolins, and proteoglycans.^[^ ^62^ ^]^ Studies have shown that the tissue‐targeting capability of EVs is closely related to the components on their membranes, such as integrins and the protein corona.^[^ ^63^, ^64^ ^]^ For example, integrins on the surface of tumor‐derived EVs (TEVs) can mediate organ‐specific colonization by fusing with target cells in a tissue‐specific manner, thereby initiating the formation of pre‐metastatic niches.^[^ ^64^ ^]^ It is noteworthy that the uptake of EVs by recipient cells does not necessarily imply the achievement of functional delivery. Although this is often desired, the internalized EVs may also be released back into the extracellular environment.^[^ ^65^ ^]^

## 3 Effects of TEVs on Tumor Immune Evasion

**Figure 2:**
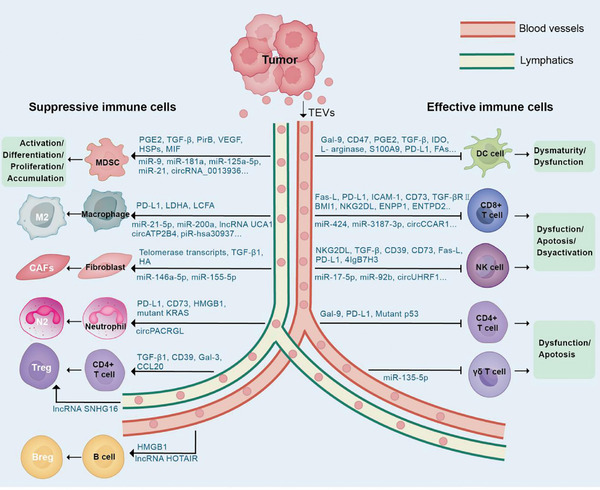
The effects of tumor‐derived extracellular vesicles in immune regulation. TEVs transfer their cargo to reprogram the recipient immune cells to promote an immunosuppressive TME.

TME is composed of heterogeneous populations of non‐malignant cells that interact with one another and also with the cancer cells to support tumor development and progression.  TEVs are increasingly recognized as crucial elements that regulate the interaction between tumor cells and their surroundings in the TME.  Numerous pieces of evidence show that TEVs can alter the differentiation of macrophages and myeloid precursor cells, interfere with dendritic cell differentiation and maturation, attenuate the cytotoxicity of NK cells, suppress T cell responses, activate cancer‐associated neutrophils and cancer‐associated fibroblasts, and induce regulatory T and B cells.^[^ ^66^, ^67^ ^]^  In the following section, we outline the mechanisms by which TEVs facilitate tumor immune escape by interacting with different immune cells in the TME (**Figure** **2**).

### 3.1 TEVs Promote Immunosuppressive Phenotypes in Macrophages and Facilitate Tumor Immune Escape

Tumor‐associated macrophages (TAMs) represent an important component of tumor infiltrating immune cells.  Upon the stimulation of different cytokines, TAMs are polarized into two functionally distinct forms, namely M1 and M2, to modulate the host immune system against tumors.^[^ ^68^ ^]^  M1 macrophages are classically activated by IFN‐γ or lipopolysaccharide, which promote inflammation and inhibit tumor growth.  On the other hand, M2 macrophages are alternatively activated by IL‐4, IL‐10, or IL‐13, which suppress inflammation and support tumor growth.^[^ ^68^ ^]^  Numerous studies have shown that M2 macrophages reduce antigen presentation ability, weaken the ability to destroy tumor cells, and release immunosuppressive factors. Additionally, they induce tumor angiogenesis, promote pre‐metastatic niche formation, and regulate T‐cell function, thereby driving tumor progression.^[^ ^69^, ^70^ ^]^  To this end, TEVs have been shown to influence the equilibrium of diverse functional subsets of macrophages, promoting M2 polarization and thus establishing an immunosuppressive tumor microenvironment (Figure 2).^[^ ^71^, ^72^, ^73^ ^]^

Over the past few decades, a large number of studies have identified functional RNAs carried by TEVs, including miRNA (microRNA), lncRNA, circRNA, and piRNA (PIWI‐interacting RNA) (**Table** **1**).  For example, Yin et al. have demonstrated that miR‐21‐5p and miR‐200a, encapsulated within extracellular vesicles derived from colorectal cancer, mediate immune suppression.^[^ ^74^ ^]^  Mechanistically, these miRNAs synergistically induce the immunosuppressive phenotype and PD‐L1 expression in macrophages by targeting the PTEN/AKT and SCOS1/STAT1 pathways, thereby reducing the activity of CD8+ T cells.^[^ ^74^ ^]^  Interestingly, research has found that TEVs can transmit ceRNA (competing endogenous RNA) to modulate miRNA activity, thereby influencing macrophage polarization.  Wu et al. identified that EVs derived from oral cancer stem cells are enriched in the lncRNA UCA1, which can modulate the PI3K/AKT pathway in macrophages by interacting with miR‐134.^[^ ^75^ ^]^  This interaction facilitates the polarization of macrophages toward the M2 phenotype, consequently suppressing the proliferation of IFN‐γ+CD4+ T cells.^[^ ^75^ ^]^  Similarly, a recent study has shown that circRNA (such as circATP2B4) delivered in TEVs also acts as ceRNA of miRNA.^[^ ^76^ ^]^  CircATP2B4 functions as a molecular sponge to sequester miR‐532‐3p, thereby alleviating its inhibitory effect on SREBF1.  This process promotes the M2 polarization of macrophages via activating the PI3Kα/AKT signaling pathway, resulting in immune suppression.^[^ ^76^ ^]^ Moreover, in pancreatic neuroendocrine neoplasms, PIWI‐interacting RNA (piRNA), such as piR‐hsa30937, can be packed into sEVs and upregulate the expression of CD276 on TAMs via targeting PTEN to activate the PI3K‐AKT pathway.  Subsequently, CD276, as an immune checkpoint molecule, can inhibit T‐cell proliferation and reduce IFN‐γ generation, leading to immune escape.^[^ ^77^ ^]^

**Table 1:** Related cargo and mechanisms of TEV‐induced macrophage pro‐tumor subtype polarization.

Functional components	Type of cancer	Mechanism	Function	Reference
**Nucleic acid**				
Let‐7a	Melanoma	Let‐7a/insulin/AKT/mTOR signaling	Induces M2‐polarization	[81]
MiR‐125b‐5p	Melanoma	MiR‐125b‐5p/LIPA (lysosomal acid lipase A)	Induces pro‐tumor phenotype and promotes macrophage survival	[82]
MiR‐106b‐5p, miR‐18a‐5p	Breast cancer	MiR‐106b‐5p/PTEN/AKT/PD‐L1 and miR‐18a‐5p/PIAS3/STAT3/PD‐L1	Induces M2‐polarization and upregulates the PD‐L1 expression	[83]
MiR‐183‐5p	Intrahepatic cholangiocarcinoma	MiR‐183‐5p/PTEN/AKT/PD‐L1	Induces immunosuppressive macrophage	[84]
MiR‐21‐5p	Hepatocellular Carcinoma	MiR‐21‐5p/RhoB/MAPK	Induces M2‐polarization and promotes the secretion of TGF‐β and IL‐10	[85]
MiR‐92a‐3p	Gastric cancer	MiR‐92a‐3p/PTEN/ERK	Induces immunosuppressive macrophage	[86]
MiR‐3591‐3p	Glioma	MiR‐3591‐3p/JAK2/PI3K/AKT/mTOR	Induces M2‐polarization	[87]
MiR‐6794‐5p	Lung cancer	MiR‐6794‐5p/JAK1/STAT3	Induces M2‐polarization and decreases CD8+ T cells	[12]
MiR‐934	Colorectal cancer	MiR‐934/PTEN/ PI3K/AKT	Induces M2‐polarization	[88]
MiR‐21‐5p and miR‐200a	Colorectal Cancer	PTEN/AKT and SCOS1/STAT1	Induces M2‐polarization and increases PD‐L1 expression	[74]
CircRHCG	Triple‐negative breast cancer	CircRHCG/BTRC/TFEB	Induces M2‐polarization	[89]
CircATP2B4	Epithelial ovarian cancer	CircATP2B4/miR‐532‐3p/SREBF1/PI3Kα/AKT	Induces M2‐polarization	[76]
CircNEIL3	Glioma	CircNEIL3/HECTD4/IGF2BP3	Induces immunosuppressive macrophage	[90]
LncRNA UCA1	Oral squamous cell carcinoma	LncRNA UCA1/miR‐134/PI3K/AKT	Induces M2‐polarization and inhibit IFN‐γ+ CD4+ T‐cell proliferation	[75]
**Protein and lipid**				
LCFA (long‐chain fatty acids )	Lewis lung carcinoma, melanoma, hepatoma, and colon carcinoma	Reprograms lipid metabolism in macrophage	Induces M2‐polarization and suppresses CD8+ T cell function	[78]
Laminin	Ovarian cancer	Laminin/integrin αvβ5/AKT/Sp1	Induces M2‐polarization and increases the secretion of CXCL5 and CCL2	[91]
ELFN1‐AS1	Gastric cancer	ELFN1‐AS1/miR‐4644/PKM	Induces M2‐polarization	[92]
CD47	Lung cancer	CD47/SIRPα	Induces the proliferation of tumor‐associated macrophages	[93]
CXCL1	Triple‐negative breast cancer	CXCL1/EED/PD‐L1	Induces M2‐polarization and increases PD‐L1 expression	[94]
Nicotinamide	Head and neck squamous cell carcinoma	Nicotinamide/USP7/NF‐κB	Induces immunosuppressive macrophage and decreases the release of cytokines IL6 and IL8	[95]
S100A11	Osteosarcoma	S100A11/JAK2/STAT3	Induces M2‐polarization and increases the secretion of the chemokine CXCL2	[96]

In addition to transporting RNA, TEVs also harbor other regulatory molecules, including lipids and proteins (Table 1).  A recent study has found that TEVs induce macrophage M2 polarization via CD36, which is identified as a fatty acid translocase.^[^ ^78^ ^]^  Mechanistically, CD36 facilitates the phagocytosis of LCFA (long‐chain fatty acids) within TEVs by TAMs, thereby fueling TAMs and triggering their immunosuppressive functions (Table 1).^[^ ^78^ ^]^  Moreover, Gan et al. have shown that LDHA (lactate dehydrogenase A) participates in the M2 polarization of macrophages in renal cell carcinoma.^[^ ^79^ ^]^  Specifically, LDHA enhances the expression of EPHA2 (ephrin type‐A receptor 2) within TEVs.  Subsequently, EPHA2 is conveyed to macrophages via TEVs, where it activates the PI3K/AKT/mTOR signaling cascade, thereby facilitating M2 macrophage polarization.^[^ ^79^ ^]^

Besides inducing M2 polarization, impairing the anti‐tumor function of M1 macrophages can also lead to immune escape.^[^ ^80^ ^]^  Furthermore, macrophages have also been suggested to act as a relay station to display inhibitory molecules carried by TEVs, thereby mediating suppression of tumor immunity (Figure 2).  Golgi membrane protein 1 (GOLM1) is an oncoprotein highly expressed in multiple cancer types to promote cell proliferation, migration, and invasion, and inhibit apoptosis. GOLM1 is known to stabilize the PD‐L1 protein and facilitate the sorting of PD‐L1 into TEVs.  These PD‐L1‐enriched TEVs are taken up by the TAMs within the TME, thus resulting in high expression of PD‐L1 in the TAMs and subsequent inhibition of CD8+ T cells.^[^ ^38^ ^]^

Collectively, these studies demonstrate that TEVs induce the polarization of macrophages toward pro‐tumor phenotypes, which in turn facilitates tumor immune escape.  Therefore, comprehending the effects of TEVs on the differentiation of TAMs is crucial for a deeper understanding of tumor immune evasion mechanisms.

### 3.2 TEVs Suppress Maturation and Impair Anti‐Tumor Function of Dendritic Cells (DCs)

As antigen‐presenting cells, DCs can induce robust anti‐tumor immune responses by taking up, processing, and presenting tumor antigens on the cell surface to the T cells of the immune system.^[^ ^97^ ^]^  Interestingly, unlike the normal immune environment, TEVs were shown to prevent DCs maturation and impair the anti‐tumor function of DCs within the TME.^[^ ^98^ ^]^  The molecular mechanisms controlling the disruption of DCs maturation and its antitumor function by TEVs are summarized below.

The TEV‐mediated DC maturation disorder is primarily associated with the disruption of antigen perception and uptake, the inhibition of costimulatory factor expression, and alterations in cellular energy metabolism (Figure 2).  For example, TEVs carrying galectin‐9 have been shown to impair the antigen‐sensing ability of DCs by binding to TIM‐3 receptors on DCs.^[^ ^99^, ^100^ ^]^  Recently, EVs derived from nasopharyngeal carcinoma (NPC) were reported to alter the function of DCs via galectin‐9‐dependent signaling.^[^ ^101^ ^]^  Mechanistically, EVs carrying galectin‐9 were found to induce the maturation of the immunosuppressive DCs abundant in PD‐L1, PD‐L2, and CD200, subsequently promoting immune evasion.^[^ ^101^ ^]^  In addition, CD47 is a signaling protein expressed on tumor cells and it transmits a “don't eat me” signal to protect tumor cells from phagocytosis by immune cells.  It has been reported that TEVs enriched in CD47 could inhibit the phagocytosis of tumor cells by DCs via disrupting the interaction between tumor cells and SIRP α on DCs.^[^ ^102^, ^103^, ^104^ ^]^  Moreover, glycolytic enzymes carried on TEVs can increase ATP and lactate levels in the TME and regulate DCs’ maturation.^[^ ^105^, ^106^, ^107^ ^]^  TGF‐β is known to exhibit a dual role in tumor immunity, and overexpression of TGF‐β can lead to immune suppression.^[^ ^108^, ^109^ ^]^  TEVs carrying TGF‐β were found to induce the upregulation of TGF‐β in DCs, which further increases TGF‐β expression by forming a positive feedback loop, thereby inhibiting anti‐tumor immunity.^[^ ^110^, ^111^ ^]^

Mature DCs are capable of activating T cells via antigen presentation, thereby enhancing the initiation of antitumor immunity.  To this end, numerous studies have reported the pivotal roles of TEVs in inducing DC dysfunction within the TME.  Indoleamine 2,3‐dioxygenase (IDO) and L‐arginase are potent immunosuppressive enzymes that could be delivered by TEVs to DCs. The two enzymes promote the degradation of tryptophan and arginine, thus impeding DCs’ ability to activate T cells.^[^ ^112^ ^]^  Similarly, S100A9 protein enriched in TEVs has also been shown to reduce the levels of costimulatory molecules (CD83 and CD86) on DCs, thereby weakening their cross‐activating T cell function.^[^ ^113^, ^114^ ^]^  Moreover, PD‐L1 encapsulated in TEVs can interact with PD‐1 on the DC membranes and induce DC dysfunction.^[^ ^115^, ^116^, ^117^, ^118^ ^]^  Furthermore, PGE2 and TGF‐β delivered by TEVs were also reported to induce the upregulation of CD39 and CD73 on DCs.^[^ ^119^ ^]^  CD39 and CD73 were known to work together to produce adenosine (ADO) and suppress anti‐tumor immunity.^[^ ^120^ ^]^  Thus, PGE2 and TGF‐β carried by TEVs can induce the impairment of antigen‐presentation activity of DCs.  In addition to proteins and nucleic acids, TEVs carrying fatty acids (FAs) also impair DC function by inducing lipid aggregation within DCs and activating oxidative phosphorylation to alter the energy metabolism of DC cells.^[^ ^121^, ^122^ ^]^

### 3.3 TEVs Activate Myeloid‐Derived Suppressor Cells

MDSCs represent a heterogeneous population of immature myeloid cells with potent immunosuppressive effects. They have the same origin as DC and other immune cells, but display distinct features including immature phenotypes and morphologies and relatively weaker phagocytic activities.  MDSCs are characterized by their abilities to suppress immune responses and shield tumor cells from the host immune attack.^[^ ^123^, ^124^ ^]^  TEVs are known to regulate the tumor‐promoting effect of MDSCs primarily in two ways: i) promoting the differentiation of immature myeloid cells (IMCs) toward MDSCs while inhibiting their differentiation toward the anti‐tumor immune cells; ii) enhancing the immunosuppressive function of MDSCs.^[^ ^125^ ^]^

Numerous studies have shown that nucleic acids encapsulated in EVs, particularly the regulatory non‐coding  RNAs, can alter the proliferation and cellular functions of the recipient cells (Figure 2).^[^ ^126^ ^]^  For instance, in breast cancer with high IL‐6 expression, Jiang et al. have found that miR‐9 and miR‐181a, encapsulated within TEVs, facilitate the generation of early‐stage MDSCs.^[^ ^127^ ^]^  They elucidated that miR‐9 and miR‐181a respectively target SOCS3 and PIAS3, subsequently activating the JAK/STAT signaling pathway and promoting early‐stage MDSCs expansion, leading to robust suppression of T‐cell immunity.^[^ ^127^ ^]^  Moreover, miR‐125a‐5p contained within TEVs derived from melanoma cells modulates the phenotype and function of myeloid cells through the NF‐κB‐dependent signaling pathway, resulting in the accumulation of MDSCs and inhibition of T cell proliferation.^[^ ^128^ ^]^  Similarly, in oral squamous cell carcinoma, hypoxic TEVs containing miR‐21 were shown to activate MDSCs via targeting PTEN to upregulate PD‐L1 levels, thereby inhibiting γδ T cells.^[^ ^129^ ^]^  A few other miRNAs, such as miR‐10a, miR‐21, miR‐1260a, miR‐494‐3p and miR‐107, were also delivered by TEVs to MDSCs and promote their expansion, subsequently leading to immunosuppression (**Table** **2**).^[^ ^130^, ^131^, ^132^ ^]^  Furthermore, in bladder cancer, Shi et al. demonstrated that exosomal circRNA_0013936 enhances the production of immunosuppressive molecules in polymorphonuclear MDSCs through sponge‐like sequestration of miR‐320a and miR‐301b‐3p.^[^ ^133^ ^]^  FATP2 (fatty acid transporter protein 2) and RIPK3 (receptor‐interacting protein kinase 3) are pivotal molecules involved in the immunosuppressive function of polymorphonuclear MDSCs, ultimately inhibiting the functions of CD8+ T cells and the secretion of IFN‐γ by CD8+ T cells.^[^ ^133^ ^]^

**Table 2:** Related cargo and mechanisms of TEV‐induced MDSCs activation and proliferation.

Functional components	Type of cancer	Mechanism	Functions	Reference
**Protein**				
PGE2 and TGF‐β	Murine mammary adenocarcinoma	PGE2/cAMP TGF‐β/β‐catenin	Induce the differences of MDSCs	[134]
LILRB2/Pirb	Murine glioblastoma	PirB/LILRB2/ANGPTL8	Induces the differentiation and proliferation of MDSCs	[135]
HSP60	Colorectal cancer	HSP60/TLR2/MYD88	Induces the accumulation of MDSCs	[138]
HSP70	Renal cell carcinoma	HSP70/TLR2/MYD88	Induces the expansion and activation of MDSCs and suppresses CTL	[137, 140]
MIF	Pancreatic Cancer	MIF/CXCR2,4/CD74	Induces the differentiation of MDSCs	[139]
VEGF	Melanoma	VEGF/VEGFR2	Induces the proliferation of MDSCs	[136]
**Nucleic acid**				
MiR‐125a‐5p	Melanoma	MiR‐125a‐5p/NF‐κB	Induces the proliferation of MDSCs	[128]
MiR‐10a and miR‐21	Glioma	MiR‐10a/RORA/IκBα/NF‐κB and and miR‐21/PTEN/ PI3K/AKT	Induce the expansion and activation of MDSCs	[131]
MiR‐1246	Glioma	MiR‐1246/DUSP3/ERK	Induces the differentiation and activation of MDSCs	[141]
MiR‐21	Oral squamous cell carcinoma	MiR‐21/PTEN/PD‐L1	Induces the activation of MDSCs and inhibits γδ T cells	[129]
MiR‐1260a and miR‐494‐3p	Pancreatic ductal adenocarcinoma	Alter intracellular calcium and glycolysis in a SMAD4‐dependent manner	Induce the expansion of MDSCs	[132]
MiRNA‐107	Gastric cancer	MiRNA‐107/PTEN/PI3K/ARG1 and miRNA‐107/DICER1	Induces the expansion and activation of MDSCs	[130]

In addition, TEVs are also known to harbor a wide variety of regulatory proteins, which control the proliferation, survival, and functional properties of MDSCs (Table 2).  Xiang et al. demonstrated that TEVs containing PGE2 and TGF‐β can induce the differentiation of bone marrow myeloid cells into MDSCs, thereby promoting the secretion of various immunosuppressive molecules (including IL‐6, COX2, and arginase‐1).^[^ ^134^ ^]^  Recently, in mouse glioblastoma models, Wu et al. demonstrated that PirB contained in sEVs induces the formation of MDSCs and the construction of immunosuppressive TME.^[^ ^135^ ^]^  Another recent study has shown that TEVs significantly stimulate the proliferation of MDSCs through delivering angiogenic factors, such as VEGF (vascular endothelial growth factor), thereby facilitating immune evasion.^[^ ^136^ ^]^  Similarly, other immune regulatory molecules, including HSP60, HSP70, and MIF (migration inhibitory factor) also induce the activation of MDSCs (Table 2).^[^ ^137^, ^138^, ^139^ ^]^  These findings suggest that a substantial proportion of molecules within TEVs play a crucial role in mediating the interaction between tumor cells and MDSCs, thereby offering novel targets for preventing tumor immune evasion.

### 3.4 TEVs Endowed a Pro‐Tumor Phenotype to Neutrophils

Neutrophils are the most abundant immune cells in the body and they play a vital role in the innate immune response to defend against infection.  In cancer biology, neutrophils are manipulated by tumors to support or retard tumor growth depending on the status of the cytokine pool in the TME.^[^ ^142^ ^]^  Numerous studies suggested that neutrophils significantly influence the TME in different cancer types.^[^ ^18^, ^143^, ^144^, ^145^ ^]^  Zhang et al. reported that gastric cancer‐derived EVs could prolong survival and increase the expression of various inflammatory factors in neutrophils.^[^ ^146^ ^]^  Mechanistically, the TEVs are enriched with high mobility group protein B1 (HMGB1) and they were transported to the neutrophils, thus activating the NK‐κB pathway by interacting with the toll‐like receptor 4 (TLR4) and promoting a prosurvival autophagic response in the neutrophils (Figure 2 and **Table** **3**).^[^ ^146^ ^]^  Interestingly, the neutrophils were converted to a pro‐tumor phenotype (also known as N2‐polarized neutrophils) and promoted gastric cancer cell migration.^[^ ^146^ ^]^  On the other hand, Shi et al. reported that HMGB1‐encapsulated TEVs also targeted STAT3 and increased the levels of PD‐L1 in neutrophils, thereby suppressing the immune responses of T cells and leading to immune evasion.^[^ ^147^ ^]^

**Table 3:** Related cargo and mechanisms of TEV‐induced the proliferation and recruitment of TANs, CAFs, Tregs, and Bregs.

Functional components	Type of cancer	Mechanism	Function	Reference
**TANs**				
HMGB1	Gastric cancer	HMGB1/TLR4/NF‐кB	Induces N2‐polarization	[146]
Mutant KRAS	Colorectal cancer	p‐STAT/p‐AKT/p‐ERK	Induces the recruitment of neutrophil and increases the secretion of IL‐8	[148]
CircPACRGL	Colorectal cancer	CircPACRGL/miR‐142‐3p/TGF‐β1 and CircPACRGL/miR506‐3p/TGF‐β1	Induces N2‐polarization	[149]
**CAFs**				
Telomerase transcripts	Acute T cell leukemia	Alters microRNA transcriptome profile	Induces the proliferation of CAFs	[151]
Let‐7a‐5p	Non‐small cell lung cancer	Let‐7a‐5p/TGF‐β	Induces fibroblasts convert into CAFs	[152]
MiR‐146a‐5p and miR‐155‐5p		MiR‐146a‐5p/ZBTB2/NF‐κB and miR‐155‐5p/SOCS1/JAK/STAT3	Induces fibroblasts convert into CAFs	[14]
**Tregs**				
TGF‐β1	Colorectal cancer and gastric cancers	TGF‐β/SMAD and TGF‐β/SAPK	Induces the proliferation of Tregs	[153, 154]
CCL20	Nasopharyngeal Carcinoma	CCL20/CCR6	Induces the differentiation and recruitment of Tregs.	[155]
CD39	Non‐small‐cell lung cancer	CD39/AMPK	Induces the proliferation of Tregs	[156]
**Bregs**				
HMGB1	Hepatocellular carcinoma	HMGB1/TLR2/4 /MAPK	Induces the proliferation of TIM‐1+ Bregs	[157]
LncRNA HOTAIR	Colorectal cancer	LncRNA HOTAIR/PKM2/STAT3/PDL1	Induces the proliferation of PD‐L1+ Bregs	[158]

A recent research found that KRAS mutant colorectal cancer (CRC) cells deliver mutant KRAS to neutrophils via TEVs, inducing the recruitment of neutrophils and the secretion of IL‐8, eventually causing cancer deterioration.^[^ ^148^ ^]^  Also in CRC, Shang et al. revealed that cicrPACRGL acts as a miRNA sponge to regulate N1 neutrophil differentiation toward the N2 subtype by facilitating TGF‐β1 expression.^[^ ^149^ ^]^

Most recently, EVs derived from head and neck squamous cell carcinoma were shown to induce a pro‐tumor phenotype of neutrophils via directly delivering the immunosuppressive molecules, such as PD‐L1 and CD73.^[^ ^150^ ^]^  The transformed neutrophils were shown to mediate Treg cell aggregation and T cell dysfunction via the CD73/ PD‐L1 pathway, which collectively allows tumor cells to evade immune surveillance.^[^ ^150^ ^]^

### 3.5 Effect of TEVs on Cancer‐Associated Fibroblasts (CAFs)

In the past decades, CAFs have emerged as a crucial player in TME, involved in cancer matrix remodeling and immune crosstalk, thus forming an immunosuppressive TME.^[^ ^159^, ^160^ ^]^  Interestingly, numerous recent studies have demonstrated that CAFs can also induce tumor immune escape through TEVs.  For instance, Shang et al. found that EVs derived from CAFs containing has_circ_00 32138 (also known as CircHIF1A) can increase the expression of PD‐L1 on hepatocellular carcinoma cells, inducing cancer immune evasion.^[^ ^161^ ^]^

Tumor cells can mediate the transformation of normal fibroblasts into CAFs in various ways, including by using TEVs.^[^ ^159^, ^162^, ^163^ ^]^  For example, a recent study found that TEVs carrying telomerase transcripts reprogram fibroblasts via altering microRNA transcriptome profiles, thereby promoting the formation of CAFs (Table 3).^[^ ^151^ ^]^  In addition, research has shown that colorectal cancer‐derived EVs carrying miR‐146a‐5p and miR‐155‐5p activate CAFs via the JAK2–STAT3/NF‐κB pathway, and then upregulate the secretion of inflammatory cytokines (such as IL‐6, TGF‐β, and CXCL12).^[^ ^14^ ^]^  Another study has shown that sulfonylurea receptor 1 can downregulate the levels of Let‐7a‐5p, a microRNA that targets TGF‐β receptor 1 and blocks the TGF‐β signaling.  Therefore, reducing the expression of let‐7a‐5p in TEVs also promotes the conversion of normal fibroblasts into CAFs.^[^ ^152^ ^]^

Interestingly, Yang et al. have demonstrated that EVs derived from colorectal cancer cells, which carry TGF‐β1, inhibit anti‐tumor immune responses.  Mechanistically, TGF‐β1 induces the transformation of hepatic stellate cells into the CAFs’ phenotype.  Activated CAFs subsequently recruit MDSCs to downregulate the expression of NKG2D on NK cells, thereby inhibiting NK cell cytotoxicity.^[^ ^164^ ^]^ Moreover, recent research has shown that hyaluronan (HA) present in TEVs serves as a regulatory agent, facilitating the conversion of pancreatic stellate cells into CAFs and inducing the secretion of tumor‐promoting factors, including IL‐6 and IL‐10.^[^ ^165^ ^]^  Furthermore, Wang et al. found that co‐culturing with TEVs enhances fibroblasts' secretion of the chemokine CCL1.  This increased release subsequently activates CCR8, which in turn facilitates the recruitment and differentiation of regulatory T cells (Tregs), thereby forming an immunosuppressive TME.^[^ ^166^ ^]^  These findings underscore the critical role of TEVs in reshaping the TME, thereby facilitating immune evasion and promoting tumor progression.

### 3.6 TEVs Inhibit the Recruitment, Proliferation, and Cytokine Secretion by NK Cells in the TME

As a key component of the body's innate immune system, NK cells play a crucial role in surveilling and eliminating tumor cells.^[^ ^167^ ^]^  NK cells recognize tumor cells by a few well‐established mechanisms, including i) detecting the presence of activating receptor ligands that are upregulated in cancer; ii) targeting cancer cells that have a loss of major histocompatibility complex (MHC); and iii) interacting with antibodies that bind to tumor‐specific antigens on the tumor cell surface.^[^ ^168^ ^]^  TEVs are known to modulate various biological properties of NK cells, including recruitment, proliferation, cytolytic activity, cytokine secretion, and metabolism, thereby resulting in tumor immune escape.^[^ ^169^ ^]^  The underlying machinery involved in TEV‐mediated NK cell dysfunction is summarized below (Figure 2).

NKG2D is an activating receptor expressed by NK and T cells.  NKG2D ligands (such as MHC class I‐related chain (MIC) A and MICB) are frequently overexpressed in tumor cells, but display a limited expression in normal tissues.  The binding of NKG2D (on NK cells) to its ligands (on tumor cells) triggers the release of perforin and granzyme from NK cells to cause cytotoxic lysis of tumors.  Interestingly, tumor cells can load NKG2D ligands into EVs and release them outside the cell, thus avoiding direct killing by NK cells.^[^ ^170^, ^171^, ^172^ ^]^  Moreover, TEVs carrying NKG2D ligands (such as MICA/B and ULBP (UL16‐binding protein) families) were also reported to induce the downregulation of NKG2D on NK cells, thereby resulting in immune escape.^[^ ^173^, ^174^, ^175^, ^176^ ^]^

Similarly, TEVs containing high levels of TGF‐β are capable of upregulating SMAD 2/3 phosphorylation and decreasing T‐bet transcription factor expression levels in NK cells via interact with TGF‐β receptors I/II on the cell surface (Figure 2).^[^ ^45^, ^177^ ^]^  Subsequently, these changes will reduce the expression of NKG2D on NK cells and impair their antitumor cytotoxicity.

There is also emerging evidence to show that TEVs can carry various molecules with the potential to induce the apoptosis and anergy of NK cells (Figure 2).^[^ ^17^ ^]^  For instance, ADO is an immune inhibitor that can bind with the adenosine 2A receptor (A2AR) on NK cells, thereby upregulating cAMP and inhibiting cellular function by initiating a cascade of downstream signals.^[^ ^178^, ^179^ ^]^  It has been reported that TEVs carrying CD39 and CD73 can generate ADO, and induce the dysfunction of NK‐92 cells via activating A2AR.^[^ ^180^ ^]^  Another recent study also has shown that TEVs carrying Fas‐L could induce NK cells apoptosis.^[^ ^181^ ^]^  In glioblastoma, TEVs carrying 4IgB7H3 have been shown to inhibit NK‐mediated tumor lysis.^[^ ^182^ ^]^  Furthermore, TEVs bearing PD‐L1 (also known as B7‐H1) can suppress the immune responses of activated NK cells that express PD‐1.^[^ ^183^, ^184^, ^185^, ^186^ ^]^  Moreover, a recent study found that in hepatocellular carcinoma, EVs derived from fructose‐1, 6‐bisphosphatase 1‐depleted hepatocytes can induce NK cells dysfunction via regulating their glycolytic activity.^[^ ^187^ ^]^

Besides proteins, NK cells can also be regulated by nucleic acids contained in TEVs (Figure 2).  Recently, a study demonstrated that RUNX1, a transcription factor that enhances NKG2D transcription, is a target of miR‐17‐5p, a microRNA contained in EVs derived from hepatocellular carcinoma.  Subsequent research verified that miR‐17‐5p inhibits NKG2D expression by downregulating RUNX1, thereby impairing the cytotoxic functions of NK cells.^[^ ^188^ ^]^  Hep3B hepatocellular carcinoma cell‐derived miR‐92b has been shown to be delivered to NK cells via EVs, which downregulated CD69 expression on NK cells and reduced their cytotoxicity against tumor cells.^[^ ^189^ ^]^  Moreover, Zhang et al. found that TEVs also deliver circUHRF1 (circular ubiquitin‐like with PHD and ring finger domain 1 RNA) into NK cells and induce immunosuppression.  Indeed, circUHRF1 enhances the expression of TIM‐3 by degrading miR‐449c‐5p, thereby inhibiting the secretion of IFN‐γ and TNF‐α from NK cells.^[^ ^190^ ^]^  Numerous other studies have shown that different TEVs cargoes such as miR‐23a, miR‐150‐5p, miR‐221‐5p, miR‐186‐5p, circular RNA ring finger domain 1 (circUHRF1), and lncRNA NEAT1 also play important roles in EV‐mediated immune escape via inducing NK cells dysfunction and exhaustion.^[^ ^45^, ^191^, ^192^, ^193^, ^194^ ^]^

### 3.7 TEVs Suppress the Antitumor Activities of Various Lymphocytes

#### 3.7.1 CD8 + T cells

**Figure 3:**
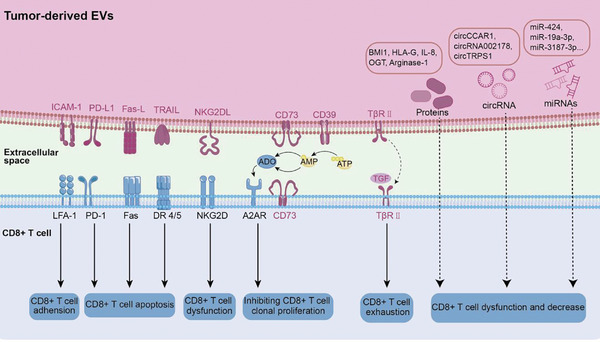
The modulation effect of TEVs on CD8+ T cell. TEVs regulate CD8+ T cells through their cargo and surface molecules. The surface molecules include Fas‐L, PD‐L1, and NKG2DL, which interact directly with the respective ligands or receptors to inhibit activation or trigger apoptosis of CD8+ T cells. TEVs can also modulate CD8+ T cell function indirectly by affecting CD39/CD73, thereby converting ATP to adenosine to suppress the antitumor cytotoxicity of CD8+ T cells. TβRII located on TEV surface can induce CD8+ T cell exhaustion by activating the TGF‐b signaling pathway. Numerous TEV cargoes also play critical roles in immune regulation.

CD8 + T cells (also commonly known as cytotoxic T lymphocytes) are pivotal players in immune defense against infection, as well as tumor cells.  Upon activation, CD8+ T cells are the direct effector cells that target and eliminate the tumor cells through recognizing tumor‐associated antigens and initiating a robust cytotoxic response.  To escape from antitumor immunity, tumor cells are shown to secrete TEVs enriched with immunosuppressive molecules to target CD8+ T cells, thereby leading to T cell exhaustion and promoting cancer progression (Figures 2 and **3**).^[^ ^195^ ^]^

Numerous functional components, which are encapsulated into TEVs, are known to modulate the immune responses of CD8+ T cells.  Notably, the non‐coding regulatory RNAs, such as miRNAs and circular RNAs, are pivotal players in these modulations.  Zhao et al. showed that the miR‐424 encapsulated in colorectal cancer‐derived EVs downregulated CD28 expression on T cells, thereby inhibiting the costimulatory signal transduction, impairing the full activation of T cells, and resulting in immune evasion (Figure 3).^[^ ^196^ ^]^  In the EVs derived from hepatocellular carcinoma, a circular RNA (circCCAR1) was shown to stabilize the PD‐1 protein on CD8+ T cells, leading to CD8+ T cell dysfunction (Figure 3).^[^ ^197^ ^]^  A few other non‐coding RNAs, including miR‐3187‐3p, miR‐19a‐3p, miR‐20a‐5p, miR‐1246, circRNA002178, circTRPS1, were also reported to cause immunosuppression by regulating CD8+ T cells (Figure 3).^[^ ^198^, ^199^, ^200^, ^201^, ^202^, ^203^ ^]^  In addition to RNAs, various proteins are also preferentially loaded in TEVs and they are reported to cause immunosuppression after being taken up by CD8+ T cells.  A recent study reported that TEVs carrying BMI1 (B‐cell‐specific Moloney murine leukemia virus integration site 1) proteins can promote cholangiocarcinoma progression by suppressing the secretion of chemokines responsible for the recruitment of CD8+ T cells.^[^ ^204^ ^]^  On the other hand, other immune regulatory molecules such as HLA‐G (human leukocyte antigen‐G), OGT (O‐GlcNAc transferase), arginase‐1, interleukin‐8, and adenosine are also specifically encapsulated in TEVs and they are shown to modulate the phenotypes, behaviors, proliferation, glucolipid metabolism, and cytotoxicity of CD8+ T cells (Figure 3).^[^ ^205^, ^206^, ^207^, ^208^, ^209^ ^]^

Interestingly, the membrane molecules of TEVs (including Fas‐L, PD‐L1, TGF‐β1, TRAIL, and CTLA‐4) also play critical roles in regulating CD8+ T cells and inducing tumor immune escape.^[^ ^11^, ^181^ ^]^ It has been demonstrated that Fas/Fas‐L interaction is a major pathway inducing tumor immune escape via the elimination of activated T cells.^[^ ^210^, ^211^ ^]^  TEVs have been shown to express Fas‐L and its interaction with Fas receptor on CD8+ T cells could lead to apoptosis of the T cells and therefore inhibition of the antitumor T‐cell response (Figure 3).^[^ ^212^ ^]^  Besides the Fas‐L/Fas axis, tumor‐associated PD‐L1 has also been shown to induce T‐cell dysfunction and exhaustion,^[^ ^213^, ^214^ ^]^ facilitate regulatory T cell activation,^[^ ^215^, ^216^ ^]^ and suppress T cell proliferation.^[^ ^217^ ^]^  In melanoma, PD‐L1 protein encapsulated in TEVs was reported to interact with PD‐1 expressed on CD8+ T cells, leading to anti‐tumor dysfunction and promoting tumor immune escape.^[^ ^218^ ^]^  Similarly, various researches also confirmed that TEVs carrying PD‐L1 can induce apoptosis and affect the cytotoxicity of CD8+ T cells in other types of tumor, such as non‐small cell lung cancer (NSCLC), triple‐negative breast cancer and Nasopharyngeal cancer (Figure 3).^[^ ^184^, ^219^, ^220^ ^]^  Moreover, similar to the NK cells mentioned above, in prostate tumor, NKG2D on CD8+ T cells can also be downregulated by TEVs carrying NKG2DL, thus inducing CD8+ T cell dysfunction and resulting in immune escape (Figure 3).^[^ ^175^ ^]^  Recently, Xie et al. found that breast cancer cell‐derived EVs can efficiently deliver type II TGF‐β receptor (TβRII) into CD8+ T cells, thus inducing CD8+ T cell exhaustion via the TGF‐β/SMAD pathway (Figure 3).^[^ ^221^ ^]^  Furthermore, cell adhesion molecules also participate in the suppression of T cells by TEVs.  The adhesion molecule intercellular adhesion molecule 1 (ICAM‐1) was found to co‐localize with PD‐L1 on TEVs.^[^ ^222^ ^]^  This exosomal ICAM‐1 was shown to interact with lymphocyte‐function‐associated antigen‐1 (LFA‐1), which is highly expressed in activated T cells.  However, the blockade of ICAM‐1 on TEVs could inhibit the interaction of TEVs with CD8+ T cells and weaken PD‐L1‐mediated suppressive effects of TEVs (Figure 3).^[^ ^222^ ^]^

In addition, EVs were further shown to deliver the respective enzymes to catalyze the generation of the immune inhibitory molecules.  Adenosine is an immunosuppressive metabolite present at high concentrations in the TME, which is generated via the catalysis of CD73/CD39.^[^ ^223^ ^]^  Wang et al. Have recently shown that glioblastoma‐derived EVs contain high levels of CD73 and they are taken up effectively by T cells to increase the CD73 expression on the T cell surface.  These T cells with CD73 upregulated could then catalyze the transformation of AMP into adenosine and activate the adenosine receptor 2A (A2AR), thereby inhibiting the clonal proliferation and cell cycle entry of T cells by interfering with their aerobic glycolysis (Figure 3).^[^ ^224^ ^]^  Interestingly, research has reported that TEVs also harbor another enzyme involved in adenosine metabolism, known as ENPP1 (extracellular nucleotide pyrophosphatase phosphodiesterase 1), or CD203a.  ENPP1 is capable of hydrolyzing 2′3'‐cGAMP, which subsequently inhibits the cGAS‐STING pathway in immune cells and reduces the infiltration of effector T cells.^[^ ^225^ ^]^  Similarly, a recent study found that the ATPase enriched in gastrointestinal cancer is ectonucleoside triphosphate diphosphohydrolase 2 (ENTPD2), but not CD39 (also known as ENTPD1).  Further studies demonstrated that ENTPD2  can be released into extracellular space in the form of EVs, thereby suppressing the CD8+ T cell response via blocking ATP‐P2×7 receptor (P2×7R)‐mediated NFATc1 activation while promoting adenosine‐A2AR signaling.^[^ ^226^ ^]^

#### 3.7.2 CD4 + T cells

Upon activation and differentiation into various effector subtypes, CD4+ T cells (also commonly known as T helper cells (Th cells)) play a crucial role in mediating immune response through the secretion of specific cytokines.  As crucial players of innate and adaptive immunity, CD4+ T cells activate cells of the innate immune system, B‐lymphocytes, cytotoxic T cells, and other nonimmune cells.^[^ ^227^ ^]^  Interestingly, numerous studies have reported that TEVs interact with CD4+ T cells and regulate antitumor immunity (Figure 2).^[^ ^75^, ^228^ ^]^

Previous investigators proposed that TGF‐β1 expressed on the membrane of TEVs can induce the proliferation of regulatory T cells (Tregs), which are also known as Foxp3‐positive CD3+CD4+ cells (Table 3).^[^ ^229^ ^]^  Recently, Huang et al. found that circGSE1 (a circRNA) within TEVs also promotes the expansion of Tregs by regulating the TGF‐β1/TGF‐β1 receptor axis.^[^ ^230^ ^]^  Moreover, Wang et al. reported that NSCLC cells selectively packaged CD39 into EVs and transferred it to CD4+ T cells to reprogram their energy metabolism.^[^ ^156^ ^]^  Subsequently, it promoted proliferation of Tregs but reduced the abundance of effector T cells, thus forming an immunosuppressive TME (Table 3).^[^ ^156^ ^]^  Gal3 (galectin 3) is an immunosuppressive protein that could bind to several receptors.  And previous research found that Gal3 also interacts with CD45, a critical component for T‐cell receptor activation.^[^ ^231^ ^]^  Interestingly, a recent study demonstrated that galectin 3 binding protein expressed on TEVs forms a complex with galectin 3 and induces the increase of IL‐10+ IL‐35+ Tregs through CD45, thereby inducing immunosuppression.^[^ ^66^ ^]^  Furthermore, Mrizak et al. have shown that nasopharyngeal carcinoma‐derived EVs secrete the CCL20 chemokine and induce the differentiation and recruitment of Tregs in the TME, thus leading to tumor immune escape.^[^ ^155^ ^]^ 

In addition, studies have shown that TEVs can impair the cytotoxicity of CD4+ T cells (Figure 2).  Epstein‐Barr virus‐infected nasopharyngeal carcinoma cells were shown to secrete EVs abundant in galectin‐9 (a ligand of the membrane receptor Tim‐3), which could induce apoptosis of CD4+ T cells via targeting Tim‐3 highly expressed on the cell surface.^[^ ^232^ ^]^  It has also been shown that TEVs carrying PD‐L1 can reduce the abundance of CD8+ and CD4+ T cells in the TME and decrease granzyme B secretion.^[^ ^233^, ^234^, ^235^ ^]^  Numerous studies have shown that mutant p53 (highly prevalent in many cancer types) is enriched in EVs and it could facilitate the formation of an immunosuppressive TME, thereby allowing tumor cells to escape from immune surveillance.^[^ ^236^, ^237^, ^238^ ^]^  Recently, Dong et al. reported that TEVs carrying the mutant p53 protein can inhibit the glucose metabolism of CD4+ T cells by downregulating some rate‐limiting enzymes (such as the platelet isoform of phosphofructokinase, hexokinase‐I and phosphorylated‐pyruvate kinase M2) of glycolysis, subsequently functionally suppressing CD4+ T cells and leading to tumor immune escape.^[^ ^239^ ^]^

#### 3.7.3 γδ T cells

Gamma delta T cells (γδ T cells) represent a unique subset of T cells that are uncommon in lymphoid organs but are particularly enriched in peripheral tissues.  Unlike the more abundant T cells (αβ T cells) in our body, which are composed of two glycoprotein chains called α and β T‐cell receptor (TCR) chains, γδ T cells possess the γ and δ TCR chains on their surface.  γδ T cells are known to promote cancer development.  In particular, γδ T17 cells could secrete IL‐17 to the TME and promote cancer growth by supporting angiogenesis.^[^ ^240^ ^]^  γδ T cells were also shown to promote the survival of myeloid‐derived suppressor cells (MDSCs) to facilitate cancer progression.^[^ ^241^ ^]^  Activated γδ T cells also participate in tumor immune surveillance.^[^ ^242^ ^]^  CD73+ γδ1 Treg cells in the TME have been reported to promote immune evasion via generating adenosine.  EVs derived from breast cancer cells containing lncRNA SNHG16 can upregulate CD73 expression on γδ1 Treg cells via targeting miRNA‐16‐5p to activate the TGF‐β/SMAD5 axis, thus inducing immunosuppression (Figure 2).^[^ ^243^ ^]^  In addition, Li et al. found that gastric cancer cell‐derived EVs can deliver miR‐135b‐5p toward Vγ9Vδ2 T cells and suppress their anti‐tumor activity.^[^ ^244^ ^]^  Mechanistically, miR‑135b‑5p was shown to downregulate the specificity protein 1, thus inducing the apoptosis of Vγ9Vδ2 T cells and reducing their secretion of cytotoxic cytokines (such as TNF‐α and IFN‐γ) (Figure 2).^[^ ^244^ ^]^

#### 3.7.4 B Lymphocytes

B cells (also called B lymphocytes) are mononuclear lymphoid cells that are involved in the adaptive immune response and play a pivotal role in humoral immunity through antigen presentation and antibody production.  There are numerous pieces of evidence that B cells play important roles in regulating the occurrence and development of tumors.^[^ ^245^ ^]^  The immunosuppressive regulatory B (Breg) cells are well known to be detrimental to antitumor immunity in the TME through the secretion of various anti‐inflammatory cytokines (Figure 2).^[^ ^246^, ^247^ ^]^

In hepatocellular carcinoma, TEVs containing HMGB1 were shown to interact with the Toll‐like receptor 2/4 and induce the proliferation of TIM‐1+ Breg cells, thereby promoting tumor immune evasion (Table 3).^[^ ^157^ ^]^  Similarly, Xie et al. found that the lncRNA HOTAIR contained in colorectal cancer cell‐derived‐EVs can induce B cells to acquire regulatory features, thereby suppressing anti‐tumor immunity.  Mechanistically, lncRNA HOTAIR upregulates PD‐L1 expression on B cells by inhibiting the degradation of pyruvate kinase M2 (Table 3).^[^ ^158^ ^]^  Recently, pancreatic ductal adenocarcinoma (PDAC)‐derived EVs were shown to inhibit PDAC serum‐mediated complement‐dependent cytotoxicity toward cancer cells.^[^ ^248^ ^]^  Tumor‐associated antigens carried on the surface of TEVs were shown to induce autoantibodies in patient serum and exert a decoy function against complement‐dependent cytotoxicity.^[^ ^248^ ^]^

## 4 Treatment Strategies to Reversing Immune Escape

Although immunotherapy and chemotherapy exhibit substantial antitumor efficacy in certain patients, leading to long‐term survival, a significant proportion of patients remain resistant to these treatments.^[^ ^249^, ^250^ ^]^  This resistance suggests the existence of underlying immunosuppressive mechanisms that facilitate immune evasion in non‐responsive tumors.^[^ ^251^ ^]^  Consequently, the tumor immune escape mechanism mediated by TEVs offers novel insights for developing alternative therapeutic approaches.  Interestingly, because of EVs' inherent low immunogenicity, high stability, and possible stimulation of anti‐tumor immune responses, EVs have been utilized to deliver therapeutic agents including nucleic acids, peptides, and small‐molecule drugs.^[^ ^252^ ^]^  In the following section, several novel therapeutic strategies based on the escape mechanisms of TEVs are discussed.

### 4.1 Targeting the Production and Cargo‐Sorting of TEVs

In recent years, there have been three main treatments currently being developed to inhibit the biogenesis and cargo‐sorting of TEVs, including pharmacotherapy, genetic therapy, and clearance therapy.^[^ ^253^ ^]^

Pharmacological inhibition of TEVs has been extensively studied over recent decades. In a recent study, high expression of histone lysine‐specific demethylase 1 (LSD1) in gastric cancer was shown to suppress the antitumor effect of T cells by shedding EVs enriched with PD‐L1.^[^ ^254^ ^]^  Importantly, LSD1 deletion in the cancer cells could reduce PD‐L1 levels in the TEVs and restore the T cell anti‐tumor response.^[^ ^254^ ^]^ Therefore, the discovery of novel LSD1 inhibitors may be adopted as a new strategy to treat gastric cancer by modulating the antitumor immunity.^[^ ^255^ ^]^  Moreover, Xie et al. identified USP8 (ubiquitin‐specific peptidase 8) as a key upstream regulator of the TGF‐β/SMAD pathway.^[^ ^256^ ^]^  USP8 enhances the stability of the TβRII (type II TGF‐β receptor) via ubiquitination, thereby increasing the expression of TβRII in TEVs.  They also confirmed that USP8 inhibitors could effectively decrease the population of TβRII‐positive EVs in vivo, thereby inhibiting TGF‐β/SMAD signaling and preventing tumor immune evasion.^[^ ^256^ ^]^  In another study, sulfamethoxazole has been demonstrated to markedly diminish exosomal PD‐L1 levels in tumor‐bearing mice, suggesting its potential as an adjuvant for anti‐PD‐L1 treatments.^[^ ^257^ ^]^

**Figure 4:**
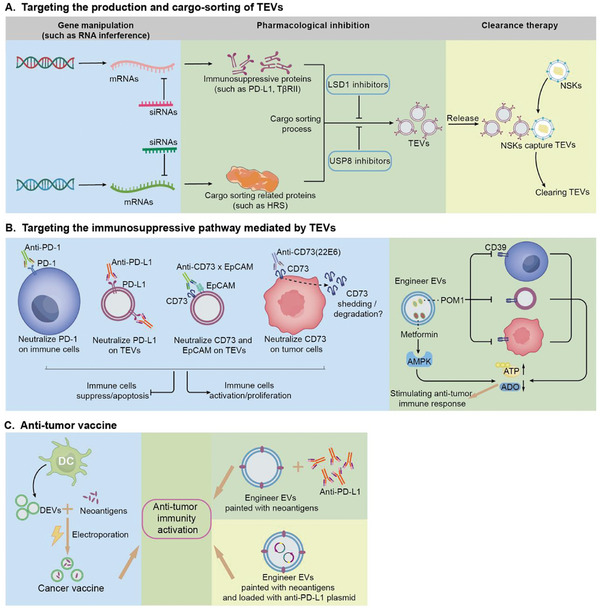
Treatment strategies to reversing immune escape. A) By targeting the production and cargo‐soring of EVs; B) By targeting the immunosuppressive pathway mediated by TEVs; (C)By utilizing anti‐tumor vaccine.

In fact, genetic manipulation has been widely used in animal experiments, and over the past decades, many experiments have validated effective targets for inhibiting the secretion of TEVs.  For example, Xiao et al. found that hepatocyte growth factor–regulated tyrosine kinase substrate (HRS), the core component of ESCRT‐0, can regulate the expression of PD‐L1 on TEVs.^[^ ^258^ ^]^  Further studies showed that the knockdown of HRS via RNA interference downregulates the secretion of TEVs carrying PD‐L1.^[^ ^258^ ^]^  Therefore, HRS is a promising target for genetic therapy, which both inhibits the release of TEVs and the expression of immunosuppressive molecules (**Figure** **4**).  In addition, a recent study demonstrated that using small interfering RNA to block the expression of immunosuppressive molecules, such as PD‐L1 and CTLA‐4, via EVs can inhibit tumor growth and immune evasion.^[^ ^259^ ^]^

Eliminating the TEVs in blood circulation is also a potential strategy to prevent tumor immune evasion.  For instance, Ye et al. developed an engineered EV, termed nanosponges and nanokillers (NSKs), by embellishing a platelet and neutrophil hybrid cell membrane on a gold nanocage surface.^[^ ^260^ ^]^  The NSKs were designed to simultaneously capture and clear circulating tumor cells and TEVs via high‐affinity membrane adhesion receptors, thus effectively blocking the TEV‐mediated cellular communication between tumor and immune cells. Moreover, these engineered NSKs can be loaded with cytotoxic drugs to give rise to additional antitumor effects.^[^ ^260^ ^]^

### 4.2 Targeting the Immunosuppressive Pathway Mediated by TEVs

The preceding section has illustrated that TEVs suppress the body's anti‐tumor immune response through the delivery of immunosuppressive factors.  Consequently, targeting the immunosuppressive pathway mediated by TEVs could represent a novel or supplementary strategy in the field of cancer immunotherapy.  Immune checkpoint blockade (ICB) is a typical representative of this treatment strategy.

The mechanism of ICB is to activate anti‐tumor immunity by blocking immune checkpoint molecules through immune checkpoint inhibitors.^[^ ^261^ ^]^  Anti‐PD‐L1 therapy, anti‐PD‐1 therapy, and anti‐CTLA‐4 therapy are some typical examples.^[^ ^262^, ^263^, ^264^, ^265^ ^]^  Interestingly, the partial influence of PD‐L1 on TEVs was only observed subsequent to the administration of anti‐PD‐L1 therapy.^[^ ^251^ ^]^

The D39/CD73/A2AR signaling cascade also plays a pivotal role in the immunosuppressive mechanisms mediated by TEVs.  Numerous studies have demonstrated that targeting effector molecules within this pathway can effectively impede tumor progression.  For example, Ploeg et al. developed a bispecific antibody (bsAb) CD73xEpCAM.^[^ ^266^ ^]^  In vitro studies demonstrated that this antibody exhibits high affinity for the surface‐labeled EpCAM on EVs and effectively inhibits CD73, thereby preventing immune suppression.^[^ ^266^ ^]^  In contrast, Kellner et al. engineered an antibody, designated 22E6, which selectively targets membrane‐bound CD73 on cancer cells and cancer cell‐derived EVs, without affecting soluble CD73.^[^ ^267^ ^]^  Notably, in a xenograft mouse model of acute lymphoblastic leukemia, treatment with 22E6 initially suppressed tumor growth.  Subsequently, all mice treated with 22E6 exhibited complete loss of CD73 expression on tumor cells, suggesting a mechanism of tumor immune evasion (such as CD73 shedding or degradation).^[^ ^267^ ^]^  In addition, Wu et al. developed a therapeutic strategy by encapsulating POM1 (a CD39 antagonist) and metformin (an AMP‐activated protein kinase (AMPK) agonist) within TEVs.^[^ ^268^ ^]^  These engineered EVs can elevate extracellular ATP levels, subsequently stimulating a synergistic anti‐tumor immune response that effectively inhibits tumor progression.  Furthermore, these nanomedicines can also reduce the accumulation of adenosine, thereby alleviating immune suppression.^[^ ^268^ ^]^

### 4.3 Antitumor Vaccine

Since the immunosuppressive TME plays a significant role in cancer progression, reshaping the TME and activating anti‐tumor immunity are crucial steps in inhibiting tumor advancement.  Tumor vaccines represent an effective approach to stimulate anti‐tumor immunity.

Interestingly, recent research has focused on the potential of EVs as components of tumor vaccines, leading to the development of therapeutic tumor vaccines based on EVs.^[^ ^269^ ^]^  A recent study found that using EVs derived from DCs as an antigen carrier can elicit potent anti‐tumor immune response, and inhibit cancer progression.^[^ ^270^ ^]^  In the study, the researchers introduced neoantigens into dendritic cell derived‐EVs (DEVs) through electroporation to create a tumor vaccine.  Meanwhile, the study also demonstrated that compared with traditional liposome vaccines, EV‐based vaccines have superior anti‐tumor efficacy due to the active protein loading.^[^ ^270^ ^]^  Moreover, in mouse cancer models, Zhang et al. demonstrated that neoantigen‐painted EV‐based vaccine combined with anti‐PD‐L1 treatment can achieve complete tumor eradication and sustainable immunological memory.^[^ ^271^ ^]^  Furthermore, Tong et al. employed TEVs as antigen sources and Akk‐OMV (outer membrane vesicle from Akkermansia muciniphila) as a natural adjuvant to formulate a novel cancer vaccine named Lipo@HEV.^[^ ^271^ ^]^  This vaccine stimulates DC maturation and induces CTL responses.  Additionally, Lipo@HEV can be loaded with plasmids for gene therapy. Upon peritumoral administration, it suppresses PD‐L1 expression, thereby mitigating the adverse effects associated with systemic administration of immune checkpoint inhibitors.^[^ ^272^ ^]^  Collectively, combination anti‐tumor vaccines and other immunotherapies have emerged as a novel strategy for treating tumors.

## 5 Clinical Trials of EV‐Based Anticancer Therapies

The previous sections have highlighted the significant potential of EVs in anticancer therapy based on extensive preclinical research. However, robust clinical validation is necessary for the successful translation from basic science to clinical application. To further explore this, we conducted a search of the ClinicalTrials.gov database to identify clinical trials investigating EV‐based anticancer therapies, which are summarized in **Table** **4**.

**Table 4:** Clinical trials using TEVs in cancer treatment.

Trial Phase	Identifier	Status	Cancer type	EVs source	Treatment strategy
I	NCT01294072	Recruiting	Colon Cancer	Plant EVs	Deliver curcumin
I	NCT03608631	Active, not recruiting	Pancreatic Adenocarcinoma	Mesenchymal stromal cells‐Derived EVs	Deliver KrasG12D siRNA
I	NCT05375604	Terminated	Hepatocellular Carcinoma, Gastric Cancer Metastatic to Liver , Colorectal Cancer Metastatic to Liver	HEK293 cells derived EVs	Deliver ASO‐STAT6
I	NCT04453046	Terminated	Squamous Cell Carcinoma of the Head and Neck	None	Eliminate immunosuppressive EVs
I	NCT05559177	Unknown status	Bladder Cancer	Dendritic cell‐derived EVs and tumor derived EVs	Antitumor vaccine
II	NCT01159288	Completed	Non Small Cell Lung Cancer	Dendritic cell‐derived EVs	Antitumor vaccine
II	NCT00042497	Suspended	Melanoma	Dendritic cell‐derived EVs	Antitumor vaccine

In the Phase I clinical trial NCT04453046, the study was terminated after 2 years, having recruited only 2 participants. Participant recruitment difficulties may have been a key factor contributing to the trial's termination. Another phase I clinical trial, NCT05375604, had assessed the efficacy of exoASO‐STAT6 in genetically matched models of colorectal cancer and hepatocellular carcinoma prior to its official initiation. ExoASO‐STAT6 is a designed exosome‐based therapeutic that delivers an antisense oligonucleotide (ASO) aimed at targeting STAT6. Kamerkar et al. demonstrated that exoASO‐STAT6 monotherapy could achieve over 90% tumor growth inhibition, with 50% to 80% of patients achieving complete remission.^[^ ^273^ ^]^ However, the trial was subsequently terminated, and the specific results have not been disclosed.

In the phase II clinical trial, NCT00042497 was terminated in 2005 and remains inactive to date, with the specific reasons for its discontinuation undisclosed. According to the database, another phase II trial, NCT01159288, evaluating exosome‐based vaccine therapy for non‐small cell lung cancer, has been completed in France; however, the results have not yet been made publicly available.

In the early stages of EVs development, research teams have preliminarily validated their safety through Phase I clinical trials. For example, Escudier et al. conducted a clinical trial to assess the feasibility and safety of dendritic cell‐derived EVs pulsed with melanoma‐associated antigen (MAGE) peptides in treating patients with advanced melanoma.^[^ ^274^ ^]^ The trial results demonstrated that patients exhibited good tolerance to the engineered EVs; however, MAGE 3‐specific CD4+ and CD8+ T cell responses were not observed in peripheral blood, and only one patient achieved a partial response. This indicates that the EVs formulation has limited efficacy in eliciting anti‐tumor immunity.^[^ ^274^ ^]^ Another clinical trial targeting non‐small cell lung cancer employed exosomes directly pulsed with MHC class I peptides. The results of this trial showed a more favorable trend in long‐term survival.^[^ ^275^ ^]^

During our search, we observed that most of the clinical trials related to TEVs in the ClinicalTrials.gov database primarily focus on their use in liquid biopsy, with relevant content already extensively reviewed.^[^ ^276^, ^277^ ^]^ Based on this, we propose a potential application of TEVs in the context of immunotherapy. For instance, the combination of TEVs with CAR‐T therapy. CAR‐T therapy is a prominent research area in immunotherapy and has demonstrated substantial clinical progress.^[^ ^278^ ^]^ Given that TEVs are crucial tools for modulating tumor‐suppressive immune cells, could they not serve as effective agents for enhancing T cell functionality, enabling the generation of CAR‐T cells capable of targeting both TEVs and tumor cells?

Overall, EV‐based anti‐tumor therapies remain in the early phases of clinical development, and their therapeutic efficacy in combating tumors requires thorough evaluation through additional clinical trial data.

## 6 Conclusion

As numerous studies have confirmed the composition, characteristics, and function of EVs, EVs have emerged as an important player in cancer biology and also antitumor immunity. These findings reveal that TEVs mediate immune escape through multiple signaling pathways and suggest potential applications of EVs in? Anti‐tumor therapy. Although numerous in vitro and in vivo research have established the critical role of TEVs in immune evasion, translating these findings into effective therapeutic strategies requires a more comprehensive understanding of the biological properties and functions of TEVs.  However, several challenges must be addressed to expedite the rapid development of EVs.^[^ ^279^ ^]^

First of all, as researchers delve deeper into the composition and functions of EVs, various EV subtypes have been identified. However, non‐vesicular extracellular particles (NVEPs) overlap with EVs in size and physicochemical properties. Current EV isolation methods have their respective limitations, making it challenging to efficiently recover specific EV subtypes(summarized in **Table** **5**).^[^ ^280^, ^281^ ^]^  In addition, processing strategies for different biological sample sources, including blood, urine, and cell culture media, require personalized adjustments. Therefore, there is a pressing need to develop novel exosome isolation techniques that offer high recovery efficiency and selectivity. The development of complementary separation techniques holds significant promise as a potential strategy to achieve this goal.^[^ ^282^ ^]^ The ISEV has proposed the standardization of sample collection, isolation, and analysis methods in EV research.^[^ ^1^ ^]^ This standardized framework could assist research teams in advancing the development and application of complementary separation techniques by integrating multiple technologies, guided by the analysis of existing literature data. Notably, microfluidics is an advanced technology that integrates functionalities such as separation and analysis.^[^ ^283^ ^]^ Laboratories with the requisite resources are encouraged to leverage microfluidic platforms for the isolation and characterization of EV subpopulations. Such efforts would enable the establishment of a comprehensive database detailing the physicochemical properties of EV subpopulations, thereby providing a robust foundation for refining existing methodologies and developing novel approaches.

**Table 5:** Major methods of EVs isolation/purification.

Method	Principle	Advantages	Limitation
Polymer precipitation	Hydrophilic or hydrophobic polymer adhering to and precipitating EVs	Low cost High recovery Time efficient	Low purity Lack of selectivity
Ultrafiltration	Separate EVs based on size and molecular weight via cut‐off filtration.	Low cost Time efficient Simple	Potential damage of EVs Membrane clogging and blockage Lack of selectivity
Tangential Flow Filtration	Separate EVs based on size and molecular weight via tangential flow filtration	High throughput Minimal sample damage Low risk of pore blocking	Equipment cost Possible contaminant co‐isolation Lack of selectivity
Differential ultracentrifugation	Separate EVs from other substances based on density and size	Appropriate for large‐volume samples Cost‐effective	May induce aggregation of EVs Low recovery Low purity Labor‐intensive
Density gradient/cushion	Isolate certain EVs from EPs based on density	High purity Compatible with diverse, well‐prepared biological samples	Low recovery Labor‐intensive Requiring extended time allocation
Size exclusion chromatography	Separate EVs from other substances based on size	Preserve biological activity High recovery	Potential co‐isolating size‐similar contaminants Low‐concentration EVs necessitate further concentration
Asymmetric flow field‐flow fractionation	Separate EVs by size with an additional flow field applied perpendicular to the flow direction	Cost‐effective High selectivity	Necessitates high‐cost instrumentation Demand comprehensive procedural fine‐tuning Low recovery
Free‐flow electrophoresis	Separate EVs based on charge in a flowing medium using a perpendicular electric field	Minimal sample damage High selectivity Simple	Necessitates high‐cost instrumentation Demand comprehensive procedural fine‐tuning Low recovery
Ion‐exchange chromatography	Separate EVs based on their charge through reversible exchange with ion exchange resin	Low mechanical stress Reproducibility No special equipment	Limited suitability for complex biofluids Optimization required
Immuno‐precipitation or affinity‐precipitation	Separate EVs based on affinity/molecular recognition	High selectivity Simple	Low recovery Elution difficulty affecting EVs integrity Antibodies employed for EVs capture lack clinical validation
Microfluidics	Microscale purification based on physical properties or biochemical characteristics of EVs	Easy automation and integration Concurrent isolation and profiling Low‐volume samples Fast processing	Necessitates high‐cost instrumentation Demand comprehensive procedural fine‐tuning Large volumes of starting materials Low sample capacity

Another challenge is the lack of universal markers for distinguishing different EVs subtypes.^[^ ^1^ ^]^  Over the past decade, numerous molecular markers, including TSG101, CD81, CD9, CD63, and ALIX, have been proposed as potential indicators to distinguish different EV subtypes. However, subsequent studies have revealed that these markers are not only expressed in various EV subtypes, but may also be present in other subcellular structures.^[^ ^1^, ^284^, ^285^ ^]^ Currently, several proteins, including Annexin A1,^[^ ^53^ ^]^ SLC3A2, and BSG for ectosomes,^[^ ^25^ ^]^ and Lamp1 for exosomes,^[^ ^286^ ^]^ have been proposed as potential markers for various EV biogenesis pathways. However, the universality and acceptance of these markers remain uncertain.^[^ ^1^ ^]^ These findings suggest the significant challenge of identifying a single marker that can reliably distinguish different EV subtypes. Therefore, in situations where markers are not yet capable of fully distinguishing between different EV subtypes, a more pragmatic approach may involve quantitatively assessing the effectiveness of various markers in isolating EV subpopulations, tailored to the specific requirements of the study.

Moreover, the limited understanding of EVs' fates in circulation restricts the analysis of their pharmacokinetics properties, thus hindering the optimization of the doses and dosing schedule of EV‐based therapy.^[^ ^9^ ^]^ In contrast to synthetic lipid‐based nanoparticles, EVs are enriched with a diverse array of bioactive molecules, which impart distinctive transport properties. Lenzini et al. have demonstrated that aquaporin‐1 (AQP1), a water channel protein present on the surface of EVs, plays an important role in regulating the deformability of these vesicles, thereby facilitating their traversal through the extracellular matrix (ECM).^[^ ^287^ ^]^ To achieve long‐range transport within the organism, EVs must traverse both the interstitial space and the bloodstream. Consequently, the nature of EV transport within these two compartments significantly influences their systemic distribution. Sariano et al. have elucidated that the interstitial flow and the engagement of the ECM with EVs are principal modulators of the mechanisms governing interstitial transport.^[^ ^288^ ^]^ In the bloodstream, the internalization of EVs by endothelial cells of both blood and lymphatic vessels is also an important factor that deserves attention.^[^ ^9^, ^289^, ^290^ ^]^ Due to the organ‐specific heterogeneity of endothelial cells and the unique molecular compositions present on their surfaces, a major challenge in developing engineered EV formulations is to prevent the overlap between the targeting moieties of the EVs and the molecules expressed on endothelial cell membranes. Such overlap may interfere with the proper recognition of target cells.^[^ ^291^ ^]^ One potential solution may involve designing EVs through gene editing to avoid interaction with endothelial cell surface molecules, or utilizing antibodies to block the relevant ligands.^[^ ^292^ ^]^

Overall, there is no doubt that the rapid advancement of this emerging field will lead to a deeper understanding of the regulatory mechanisms in multicellular organisms. To drive the development of EV‐based antitumor therapies, it will be crucial to overcome the challenges outlined above, particularly through interdisciplinary collaboration.

## Conflict of Interest

The authors declare no conflict of interest.

## Author Contributions

X.F.L. wrote the main manuscript text. Q.S.Z., K.K.T., and L.W.F. revised the manuscript. All authors read and approved the final manuscript.
